# Fatigue trajectories during pediatric ALL therapy are associated with fatigue after treatment: a national longitudinal cohort study

**DOI:** 10.1007/s00520-022-07456-x

**Published:** 2022-12-13

**Authors:** Elin Irestorm, Lindsay M. H. Steur, Gertjan J. L. Kaspers, Natasha K. A. Van Eijkelenburg, Inge M. Van der Sluis, Natasja Dors, Cor Van den Bos, Wim J. E. Tissing, Martha A. Grootenhuis, Raphaele R. L. Van Litsenburg

**Affiliations:** 1 Faculty of Medicine, Department of Paediatrics, Lund University, Lund, Sweden; 2 Princess Maxima Center for Pediatric Oncology, Utrecht, The Netherlands; 3 Department of Pediatric Oncology, Emma Children’s Hospital, Amsterdam UMC, Amsterdam, The Netherlands; 4 Department of Pediatric Oncology, Emma Children’s Hospital, Amsterdam UMC, Vrije Universiteit Amsterdam, Amsterdam, The Netherlands; 5 Department of Pediatric Oncology, Sophia Children’s Hospital, Erasmus Medical Center, Rotterdam, The Netherlands; 6 Department of Pediatric Oncology, Amalia Children’s Hospital, Radboud University Medical Center, Nijmegen, The Netherlands; 7 Department of Pediatric Oncology, University of Groningen, University Medical Center Groningen, Groningen, The Netherlands

**Keywords:** Acute lymphoblastic leukemia, Fatigue, Longitudinal studies, Survivorship, Childhood cancer, Quality of life

## Abstract

**Objective:**

Fatigue is one of the most prevalent and distressing symptoms reported by survivors of childhood cancer. There is currently a lack of longitudinal studies on cancer-related fatigue, and especially on the relationship between the course of fatigue during treatment and fatigue at follow-up. The purpose of the current study was therefore to investigate if the course of fatigue during treatment, treatment intensity, serious adverse events, sex, or age at diagnosis are associated with cancer-related fatigue after treatment.

**Methods:**

Participants were 92 children and adolescents diagnosed with acute lymphoblastic leukemia (mean age at diagnosis was 6.26 years). Fatigue was measured with PedsQL multidimensional fatigue scale proxy reports 5 months after diagnosis, 12 months after diagnosis, 24 months after diagnosis, and at follow-up 12 months after end of treatment. The effect of patient and treatment characteristics on fatigue reported at follow-up was tested through logistic regression analyses.

**Results:**

The course of fatigue during treatment significantly predicted fatigue reported at follow-up for general fatigue (*p* = .038, OR = 9.20), sleep/rest fatigue (*p* = .011, OR = 15.48), and cognitive fatigue (*p* < .001, OR = 10.78). None of the other variables were associated with fatigue at follow-up for any of the subscales.

**Conclusions:**

The findings demonstrate that fatigue reported during treatment can predict fatigue at follow-up. These results stress the need for longitudinal assessments. Healthcare professionals need to be aware that pediatric patients who are fatigued during treatment need to receive additional attention and timely interventions since cancer-related fatigue will not resolve by itself in the first year after end of treatment.

## Introduction

Cancer-related fatigue is a type of fatigue associated with either cancer or cancer treatment. It has consistently been found to be one of the most prevalent and distressing symptoms in childhood cancer survivors [1–3]. The National Cancer Institute has published recommendations for high-priority research on cancer-related fatigue in both children and adults. For survivors of pediatric cancer, these identify a need for longitudinal studies to uncover the course of fatigue over time [4]. The International Late Effects of Childhood Cancer Guidelines Harmonization Group (IGHG) recently published recommendations regarding the surveillance of fatigue in survivors of childhood cancer [5]. These recommendations include regular screenings of fatigue in survivors of childhood cancer. Furthermore, they also identify a need for longitudinal studies to investigate the change of fatigue patterns over time.

Acute lymphoblastic leukemia (ALL) is the most common type of pediatric cancer, and previous cross-sectional research has shown that survivors of ALL suffer more from fatigue at follow-up than healthy controls [6–8]. During treatment itself, patients treated for ALL also suffer more from fatigue than healthy controls [9, 10]. Treatment for pediatric ALL begins with induction therapy with intensive chemotherapy to achieve complete remission. Consolidation therapy is started after a month. These intensive treatment blocks are followed by intensification chemotherapy. After that, most patients receive maintenance therapy for 2 years after initial diagnosis to prevent relapse. Maintenance is a relative stable phase in which most patients resume their normal daily activities. Nevertheless, in the Dutch protocol, maintenance therapy includes cyclic glucocorticoids treatment in the majority of patients. Glucocorticoids, and particularly dexamethasone, are known for their neurobehavioral side effects [11]. Dexamethasone is known to affect the level of fatigue in patients treated for ALL, with patients experiencing more fatigue during periods on dexamethasone than off dexamethasone [9, 12]. However, it is still unclear whether the effect on fatigue during treatment remains after end of treatment. Little is known about how fatigue transitions from on treatment to off treatment, and more research is needed regarding which patients remain fatigued after the end of treatment. The etiology of cancer-related fatigue is not fully understood yet and is likely to be the result of an interaction of factors, where age at diagnosis and sex have been described as possible modulation factors [13]. Fatigue during treatment has been considered as an aspect of the toxicity of chemotherapy [14]. The IGHG recommendations include screening for underlying conditions that may cause fatigue, including conditions that can be effects of treatment [5].

There is currently a lack of research regarding the relationship between fatigue experienced during treatment and fatigue reported at follow-up in patients treated for ALL. Given that the survivors themselves consider fatigue to be a major cause of suffering, there is a need to better recognize who is at increased risk of fatigue in order to facilitate early recognition and intervention. Therefore, this study aimed to investigate if the course of fatigue during treatment, treatment intensity, serious adverse events during treatment, sex, or age at diagnosis is associated with fatigue 1 year after end of treatment.

## Methods

### Participants

The results described here are part of the SLAAP [*SLEEP*] study (*SL*eep in children with *A*cute lymphoblastic leukemia *A*nd their *P*arents), which is an observational, longitudinal, multicenter study on sleep, sleep–wake rhythms, quality of life, and cancer-related fatigue in pediatric ALL patients and functioning of their parents [9, 10]. Patients were identified through the Dutch Childhood Oncology Group registry that includes all pediatric patients with a diagnosis of cancer in the Netherlands. Patients were eligible if they were (1) diagnosed with primary ALL and treated according to the Dutch national first-line treatment protocol ALL-11, open to patients aged 1 to 19 years, (2) ≥ 2 years of age at first assessment, and (3) had participated in the follow-up assessment 1 year after end of treatment. Exclusion criteria were (1) if either parent or patient did not master Dutch sufficiently to complete the questionnaires and (2) if the patient was confirmed as Ikaros-positive. According to the Dutch ALL-11 protocol, treatment for Ikaros-positive patients (IKZF1 gene deletions) in the medium-risk group is 36 months instead of 24 months, and the last assessment for these patients was therefore conducted at a time point when they were still receiving treatment.

Patients were recruited in the former Dutch pediatric oncology centers: Emma Children’s Hospital/Academic Medical Center and VU University Medical Center Amsterdam, Wilhelmina’s Children’s Hospital/University Medical Center Utrecht, Sophia Children’s Hospital/Erasmus Medical Center Rotterdam, Beatrix Children’s Hospital/University Medical Center Groningen, and Amalia Children’s Hospital/Radboud University Medical Center Nijmegen, as well as from the current national center Princess Máxima Center for pediatric oncology in Utrecht.

A total of 225 patients were invited to participate in the SLAAP study. Informed consent was provided for 151 patients, out of which 127 patients were enrolled. Out of these, 92 participants were included in the final analysis for this study. Sample size calculation of the SLAAP study was based on the primary outcome sleep. No additional sample size was calculated for the outcomes presented here. Regarding non-participants, 18 were not included in the statistical analysis due to missing/incomplete data, 1 died during the study, and 16 dropped out. Age and sex were not statistically different between participants and non-participants and those not invited for the study [10].

### Procedure

**Fig. 1:**
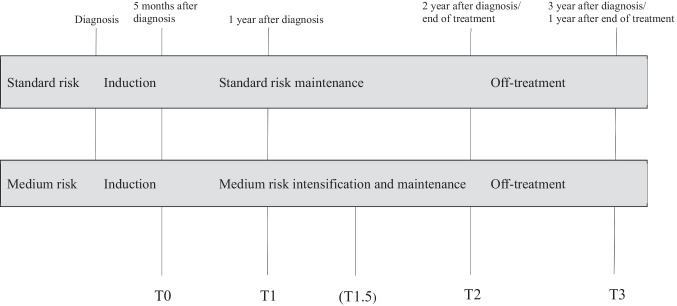
Overview of the ALL-11 treatment protocol and the four study assessments. Medium risk patients were given a fifth assessment (T1.5) during treatment with dexamethasone

Fatigue was assessed at 4 time points: 5 months after diagnosis (T0), 12 months after diagnosis (T1), 24 months after diagnosis (T2), and 12 months after end of treatment (T3). Details regarding the assessment time points are described in Fig. 1. The first assessment (T0) was planned during consolidation therapy. The second measurement (T1) was 1 year after diagnosis, during maintenance therapy. Based on response to treatment and cytogenetics, patients were stratified to the following risk groups: standard-risk group, medium-risk group, and high-risk group. During the maintenance phase, ALL treatment intensity depends on the risk group stratification. Standard-risk maintenance therapy consisted of daily oral mercaptopurine and weekly oral methotrexate. Maintenance therapy for medium-risk group patients consisted of 21-day cycles including vincristine on day 1 of each cycle, weekly oral methotrexate, continuous oral mercaptopurine, and dexamethasone orally on days 1–5. In addition, medium-risk patients received intrathecal chemotherapy around once per 4 months. Assessments were planned during a week without intrathecal chemotherapy and without dexamethasone. The last two measurements were two (T2) and three (T3) years after diagnosis, respectively. An additional assessment point was included for medium-risk participants (T1.5). This assessment was conducted during treatment with dexamethasone, and for the comparability with the standard-risk group, the data from this assessment was not included in any statistical model.

### Measures

Information on time since diagnosis, risk group stratification, Ikaros-status, and serious adverse events was collected through the Dutch Childhood Oncology Group registry. Serious adverse events are defined in the Dutch ALL-11 protocol as events that are fatal or life-threatening, require prolonged hospitalization (for grade IV infections according to the NCI-CTC classification version 4, or invasive fungal infections, severe soft tissue infections, unexpected grade III/IV serious adverse events), result in persistent or significant disability/incapacity, or are medically significant. At the baseline assessment (T0), parents filled out a survey to collect information regarding patient and parental age and sex, as well as highest parental education. The latter was classified as a binary variable, either as low-middle or higher education.

The Dutch parent-proxy version of the PedsQL™ multidimensional fatigue scale (PedsQL MFS) was used to assess cancer-related fatigue. The instrument is validated and demonstrates good psychometric properties, and Dutch references are available for healthy populations divided by age and sex [15, 16]. Furthermore, the instrument is recommended for the assessment of fatigue in children and adolescents treated for childhood cancer [5, 13]. This 18-item questionnaire allows for an overall fatigue score and three subscale scores: general fatigue, sleep-rest fatigue, and cognitive fatigue. The occurrence of problems is assessed over the past week on a 5-point Likert scale. Items are rescored to a 0–100 scale. A higher score indicates less fatigue. The questionnaire was completed paper–pencil or through a secured online web portal depending on parent preference. For the statistical analysis, only the three subscales were used as these represent different aspects of fatigue and the average of them was not considered meaningful for a longitudinal analysis. The parent-proxy reports were chosen over self-reports in order to enable consistent comparisons between age groups. Due to the low inclusion age in this study, self-reports would only have been available for a small portion of the sample. Furthermore, the use of parent-proxy reports also allowed for measurement when some participants were acutely ill during treatment.

### Statistical methods

Comparisons between the study participants who completed the final assessment and the drop-out group were made with Student *t* test for continuous variables and χ2 for categorical variables.

Cut-offs for the PedsQL MFS were calculated based on the Dutch norms for healthy controls [16]. As there are only norms for ages 2–18 for the parent-proxy reports, for those 9 participants who were over 18 at T3, the norms for 18-year-olds were used. A score more than 1.5 standard deviations (SD) below the norm mean for the corresponding age group was considered to be an indicator of fatigue. The cut-off of − 1.5 SD was based on previous research on cancer-related fatigue [6, 17, 18]. The dichotomous results from assessment T3 were used as outcome variables, with the course of fatigue during treatment, sex, risk group, serious adverse event, and age at diagnosis as independent variables. Serious adverse event was coded as 0 (no serious adverse event during treatment) or 1 (1 or more serious adverse event). The relationship between parental education and reported fatigue was explored in a univariate analysis. This variable was not included in the total model since it was based only on the highest educational level of the family’s main wage earner, which did not always correspond to the person filling out the questionnaires. Current profession and the educational level of a possible second wage earner were not available.

For each subscale of the PedsQL MFS, patients were divided into three different groups depending on their course of fatigue during treatment (assessments T0–T2): (1) fatigued at all available assessments, (2) changing fatigue status, and (3) never fatigued. To increase power and enable statistical analysis, the participants that were fatigued at all assessments and those with changing fatigue status were combined, thus creating a dichotomous independent variable. This dichotomous variable created for each of the three sub-types of fatigue. The relationship of patient and treatment characteristics with fatigue at follow-up was tested through logistic regression analyses. A regression model was formed for each subscale of the PedsQL MFS. All analyses of data were conducted using SPSS version 26.0.

## Results

Drop-out rate from enrolment to follow-up did not differ depending on risk group, age at diagnosis, or sex. Out of the 92 participants, 23 (25%) were treated according to the ALL-11 standard-risk protocol and 69 (75%) according to the medium-risk protocol. Only one participant treated according to the high-risk protocol was originally enrolled but did not complete the assessment at T3. This patient was therefore not included, and there were hence no participants treated with a high-risk protocol. Regarding sex distribution, 52 participants were male, and 40 were female. A total of 78 serious adverse events were reported in 31 (34%) different participants. The most reported serious adverse event was a life-threatening or complicated infection. The mean age at diagnosis was 6.26 years (SD 4.33), and the median age was 4.88 years. The mean age at T3 was 9.23 years (SD 4.35), and the median age was 7.31 years. Regarding parental educational level, the majority of families had at least one parent with a high education (70%).

**Fig. 2:**
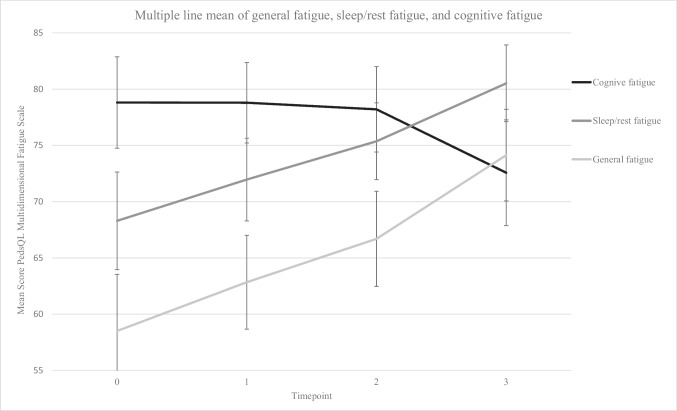
Graph of changes in parent-proxy reported fatigue over the course of treatment. Error bars: 95% confidence interval. Confidence intervals and means are reported for the whole group

Figure 2 illustrates the course of raw fatigue scores over time. For general fatigue and sleep/rest fatigue, the scores increased over time (indicating less problems at follow-up), while they decreased for cognitive fatigue (indicating more problems at follow-up). At the follow-up assessment T3, 26% of patients experienced general fatigue, 16% sleep/rest fatigue, and 22% cognitive fatigue. Regarding the course of fatigue over time, patients frequently changed status during treatment for general fatigue and sleep/rest fatigue, whereas it was less common for cognitive fatigue. The groups are described in Table 1.

**Table 1:** Description of the development of fatigue reported during treatment and at follow-up

	General fatigue	Sleep/rest fatigue	Cognitive fatigue
Course of fatigue during treatment	*n* (%)	*n* (%)	*n* (%)
Never fatigued	20 (22%)	40 (43%)	75 (82%)
Fatigued	72 (78%)	52 (57%)	17 (18%)
* Always fatigued*	*22 (24%)*	*8 (9%)*	*2 (2%)*
* Changing status*	*50 (54%)*	*44 (48%)*	*15 (16%)*
Outcome at follow-up			
Not fatigued	68 (74%)	77 (84%)	72 (78%)
Fatigued	24 (26%)	15 (16%)	20 (22%)

Course of fatigue consisted of assessments at T0, T1, and T2. Cut-off was 1.5 standard deviation below the mean for healthy controls

*n* = 92

The course of fatigue during treatment was the only independently significant predictor for experiencing fatigue at follow-up. Neither sex, risk group, nor age at diagnosis contributed to the model for any of the three subtypes of fatigue. The results from the logistic regression are presented in Table 2. For general fatigue, patients who experienced fatigue during treatment were 9.20 times as likely (*p* = 0.037, 95% CI 1.15–73.93) to experience fatigue at follow-up. The corresponding odds ratios for sleep/rest fatigue were 15.48 (*p* = 0.011, 95% CI 1.90–126.27) and for cognitive fatigue 10.79 (*p* < 0.001, 95% CI 3.12–37.26). Since the variable fatigue during treatment could have confounded the results for age and sex, a model was constructed without the fatigue during treatment as a predictor. However, neither age at diagnosis nor sex was a significant predictor for the outcome at T3 for any of the 3 subscales. Neither was parental educational level related to the reported outcome in the separate, univariate analysis.

**Table 2:** Factors associated with fatigue at follow-up 12 months after end of treatment

	*B*	SE	Sig	OR	95% CI for OR
General fatigue					
Included					
Course of fatigue	2.22	1.06	.037*	9.20	1.15–73.93
Sex	− 0.64	0.51	.211	0.53	0.19–1.45
Risk group	0.20	0.60	.742	1.22	0.38–3.96
Age at diagnosis Serious adverse event	− 0.01 0.10	0.06 0.53	.843 0.16	0.98 1.11	0.88–1.11 0.39–3.13
Sleep/rest fatigue					
Included					
Course of fatigue	2.41	1.07	.011*	15.48	1.90–126.27
Sex	− 0.99	0.67	.138	0.37	0.10–1.38
Risk group	0.39	0.76	.611	1.47	0.33–6.58
Age at diagnosis Serious adverse event	0.03 − 0.32	0.07 0.66	.715 .629	1.03 0.73	0.89–1.18 0.20–2.64
Cognitive fatigue					
Included					
Course of fatigue	2.38	0.63	< .000**	10.79	3.12–37.26
Sex	− 0.45	0.60	.455	0.64	0.20–2.06
Risk group	− 0.17	0.65	.796	0.85	0.23–3.04
Age at diagnosis Serious adverse event	− 0.06 − 1.14	0.07 0.63	.373 .607	0.94 0.72	0.83–1.08 0.21–2.48

*SE*, standard error; *OR*, odds ratio; *CI*, confidence interval. Higher ORs indicate greater odds of reporting fatigue at follow-up

^*^p < 0.05

^**^p < 0.01

Logistic regression, Coefficients of the model predicting whether a participant was fatigued at follow-up 12 months after end of treatment

## Discussion

This study aimed to investigate if the course of fatigue during treatment, treatment intensity, sex, or age at diagnosis can predict fatigue at follow-up 12 months after end of treatment. This was the first study to survey the development of fatigue during the entire course of treatment, and the first study to investigate if the course of fatigue during treatment is related to fatigue at follow-up. Fatigue was measured with multidimensional fatigue scales at four different time points, and the logistic regression models showed that the course of fatigue during treatment was the only significant predictor for all 3 subtypes of fatigue.

At follow-up, the prevalence of fatigue in our study was 26% for general fatigue, 16% for sleep/rest fatigue, and 22% for cognitive fatigue. A Cochrane review on severe fatigue after treatment for childhood cancer reported that severe fatigue ranged from 0 (bone cancer) to 61% (heterogeneous sample) [13]. The IGHG recommendations screened 24 studies with the prevalence of fatigue in survivors ranging from 10 to 85% [5]. For both reviews, the wide ranges in prevalence were explained by a variety in primary diagnosis and in screening instruments. Multidimensional fatigue instruments are recommended for both clinical practice [19, 20] and research [13, 21]. Comparisons should therefore be made with other studies utilizing the PedsQL MFS after treatment for ALL. The mean values for the different subscales reported in this study are comparable to those previously reported for parent-proxy ratings during [22] and at follow-up [6], respectively.

Cancer-related fatigue is considered a multifactorial process, and while its exact causal mechanisms remain unclear, it is generally assumed to be the result of a complex interaction between biological, psychological, and social factors. The child’s development can be considered a fourth factor that impacts this interaction, as it affects how fatigue is experienced and reported [4, 23]. Previous studies with mixed patient groups have reported that a large portion of the variance in fatigue can be explained by a biopsychosocial model [24]. The National Cancer Institute also identifies a need for longitudinal studies to uncover the medical, psychological, and social variables related to fatigue [4]. These longitudinal studies should also take the child’s ongoing development into account.

On a group level, our longitudinal data show that fatigue decreases during treatment for general as well as sleep-rest fatigue, which is in accordance with previous studies [25, 26]. Interestingly, there was a different pattern for cognitive fatigue. Two possible explanations for this are that patients can be growing into their deficits and that the demands on cognitive abilities increase with age or as a result of coming off treatment. On an individual level, patients often changed fatigue status. Fatigue status in adult long-term survivors of ALL has previously been reported to change over time, with 30% of survivors changing their fatigue status between two assessments [27]. Patients with fatigue during treatment were at high risk of persisting fatigue at follow-up. Odds ratios ranged between 9.20 and 15.48 for the different subtypes of fatigue. Furthermore, patients never experiencing fatigue during treatment were at lower risk at follow-up.

Biological factors such as cancer treatment intensity or time since diagnosis have been studied in mixed cancer groups, but not found to be related to fatigue [8, 28]. In a previous description of sleep–wake rhythms and cancer-related fatigue, we demonstrated that sleep–wake outcomes were associated with fatigue during periods without dexamethasone, but not during periods with dexamethasone [9]. The authors of a study on the effect of dexamethasone on fatigue and sleep concluded that dexamethasone significantly and adversely altered fatigue both during periods with and without dexamethasone [12]. This pattern is important for clinicians working with the patient group, as information on the neurobehavioral side effects of dexamethasone could help both patients and parents to cope with these effects. Our results indicate that this effect is temporary, since risk group did not affect fatigue reported at follow-up and standard-risk patients do not receive a cyclic dexamethasone treatment for a prolonged time like medium-risk patients do. The IGHG guidelines state an increased risk for fatigue in female survivors of ALL [5]. We therefore included sex in the models for all three fatigue subscales, but it did not significantly contribute to any of the three different models and neither did age at diagnosis.

Certain psychological problems have also been associated with cancer-related fatigue. A relationship between depressive symptoms and fatigue has previously been reported in children and adolescent survivors of childhood cancer [6], and depression is also closely linked to fatigue in adult survivors [29]. One study of adult survivors of childhood ALL and lymphoma reported that the level of depressive symptoms was the strongest predictor of persisting fatigue that did not decrease with time since treatment [27].

Finally, social aspects such as family factors are important to take into account, especially when using parent-proxy reports. A possible explanation for why the course of fatigue was a significant predictor for fatigue at follow-up is that parents who initially perceive their children as fatigued might continue to do so during and after treatment. Future research should therefore take parental functioning and well-being into account when investigating the development of cancer-related fatigue.

### Clinical implications

Our findings suggest that early interventions should be offered to those experiencing fatigue during treatment. However, most research regarding interventions for fatigue has been conducted in adult oncology patients. These include exercise, cognitive-behavioral therapy, and bright light therapy [21, 30–33]. In a recent study by Crabtree et al. [34], treatment with bright light therapy decreased fatigue in adolescents and young adults receiving cancer-directed therapy. Given the results from our study, more research is necessary regarding managing the fatigue experienced by participants during treatment in addition to follow-up [35]. A study on exercise and cancer-related fatigue during the final stages of therapy and the first period after end of therapy for childhood cancer reported that higher physical activity was related to less fatigue [35]. Physical activity generally increased over time, and increased physical activity was also associated with less cancer-related fatigue at follow-up. Given that children with cancer are less physically active than the average population, it is important to further explore the improvement of cancer-related fatigue through increased physical activity. Our results also show that participants never experiencing fatigue during treatment were at lower risk at follow-up. This has high clinical relevance, as it indicates that at-risk patients can be identified already during treatment according to their development of fatigue over time. Patients who are persistently fatigued or with changing fatigue status during treatment need to receive additional attention since fatigue will not resolve by itself in the first year. Healthcare professionals should be trained to monitor fatigue and provide interventions. To detect fatigue in patients treated for cancer, clinical screening programs which facilitate patient-reported outcomes can be used. In the Netherlands, this is done through the KLIK portal, where patients and their parents regularly fill out questionnaires online [36].

### Study limitations

Our study was the first to investigate the course of fatigue during the entire therapy and at follow-up and included a large sample of children and adolescents treated for ALL. In addition to a homogenous sample, only patients treated with chemotherapy were included. One limitation with this study was that almost 20% of participants were excluded due to missing data, which was probably caused by the many assessment points. However, these assessment points also made it possible to study the trajectory of cancer-related fatigue. Another limitation of this study was the use of only parent-proxy ratings of fatigue. Low interrater reliability between parent and child reports has been described in several studies using PedsQL MFS [6, 16, 37, 38]. There is also a lack of norms for the parent-proxy reports for adults, and the upper age limit for inclusion in combination with the long treatment protocol meant that a small portion of the participants was over 18 at T3. However, the low minimum age for inclusion in this study meant that many of the participants could not write and read at the time of assessment and the use of self-reports would not have been possible for a large part of the study group. Regardless of age, some participants were also too ill to complete the self-reports during the first assessment, which made the use of parent-proxy reports a necessity. Another limitation is that only standard and medium-risk participants completed the study. The results are therefore not representative for high-risk patients. Patients treated with a high-risk protocol were not excluded from participation, but a selection bias might have caused them not to be invited at the different centers. As only 5% of patients are classified as high-risk according to the Dutch ALL-11 protocol, they are also fewer than the other two groups. As treatment is much more intensive for high-risk patients, the outcomes might be different for them. As the treatment is also different from the standard and medium-risk protocol, the additional elements would warrant a different study design. Furthermore, another limitation was that parental mental health, and distress was not included in the study. As previously mentioned, future research should therefore take parental distress into account when investigating the development of fatigue.

## Conclusions

The findings reported here have implications for both care and future research. The findings demonstrate that fatigue reported during treatment can predict the outcome. Our results stress the need for multiple assessments during treatment, as a large proportion of participants changed fatigue status during the 24 months. The main conclusion is that patients who are continuously fatigued during treatment need to receive additional attention and timely interventions to mitigate the course of fatigue. Future studies should also include the relationship between reported fatigue and psychosocial functioning. Given the results from our study, more research is also needed necessary regarding possible interventions to decrease fatigue during as well as after treatment. Longitudinal studies focusing on following the participants for a longer time-period after end of treatment are also warranted.

## Data Availability

The datasets generated during and/or analysed during the current study are available from the corresponding author on reasonable request.
